# Storage of halved strawberry fruits affects aroma, phytochemical content and gene expression, and is affected by pre-harvest factors

**DOI:** 10.3389/fpls.2023.1165056

**Published:** 2023-05-31

**Authors:** Ashley Baldwin, Rakhee Dhorajiwala, Callum Roberts, Simone Dimitrova, Sarah Tu, Stephanie Jones, Richard A. Ludlow, Laura Cammarisano, Daniela Davoli, Robert Andrews, Nicholas A. Kent, Natasha D. Spadafora, Carsten T. Müller, Hilary J. Rogers

**Affiliations:** ^1^ School of Biosciences, Cardiff University, Cardiff, United Kingdom; ^2^ School of Medicine, Cardiff University, Cardiff, United Kingdom; ^3^ Department of Chemical, Pharmaceutical and Agricultural Sciences, University of Ferrara, Ferrara, Italy

**Keywords:** *Fragaria x ananassa*, postharvest, strawberry fruit, transcriptome, volatile organic compounds

## Abstract

**Introduction:**

Strawberry fruit are highly valued for their aroma which develops during ripening. However, they have a short shelf-life. Low temperature storage is routinely used to extend shelf-life for transport and storage in the supply chain, however cold storage can also affect fruit aroma. Some fruit continue to ripen during chilled storage; however, strawberries are a non-climacteric fruit and hence ripening postharvest is limited. Although most strawberry fruit is sold whole, halved fruit is also used in ready to eat fresh fruit salads which are of increasing consumer demand and pose additional challenges to fresh fruit storage.

**Methods:**

To better understand the effects of cold storage, volatilomic and transcriptomic analyses were applied to halved *Fragaria x ananassa* cv. Elsanta fruit stored at 4 or 8°C for up to 12 days over two growing seasons.

**Results and discussion:**

The volatile organic compound (VOC) profile differed between 4 or 8°C on most days of storage. Major differences were detected between the two different years of harvest indicating that aroma change at harvest and during storage is highly dependent on environmental factors during growth. The major component of the aroma profile in both years was esters. Over 3000 genes changed in expression over 5 days of storage at 8°C in transcriptome analysis. Overall, phenylpropanoid metabolism, which may also affect VOCs, and starch metabolism were the most significantly affected pathways. Genes involved in autophagy were also differentially expressed. Expression of genes from 43 different transcription factor (TF) families changed in expression: mostly they were down-regulated but NAC and WRKY family genes were mainly up-regulated. Given the high ester representation amongst VOCs, the down-regulation of an alcohol acyl transferase (AAT) during storage is significant. A total of 113 differentially expressed genes were co-regulated with the AAT gene, including seven TFs. These may be potential AAT regulators.

## 1 Introduction

Strawberries (*Fragaria x ananassa*) are a high value fruit whose production has increased from 4.5 M to almost 9 M tonnes per annum over the last 20 years (2000-2020, FAOstat). The top two producers are China and the USA, but strawberries are widely consumed and produced by over 70 countries worldwide. However, they are a highly perishable product. Strawberries are also highly valued in ready to eat fresh fruit salads, an increasing market which is of value in encouraging the consumption of fresh fruit.

Ripening in strawberries is non-climacteric (Gu et al., 2019) and progresses from green through to white, red ripe, and finally an over-ripe or senescent phase. During this process they undergo increasing softening especially during the last stages of ripening due to the changes in the structure and composition of the cell wall (Moya-León et al., 2019). This leads to fruit vulnerable to mechanical damage and the growth of spoilage microorganisms (Vicente et al., 2005). Once harvested, ripening is accelerated by dehydration stress which in turn promotes increases in anthocyanins, likely mediated *via* abscisic acid (ABA) (Chen et al., 2015). In addition, sugars appear to act synergistically with ABA during strawberry ripening (Jia et al., 2013). Autophagy has also recently been shown to contribute to strawberry fruit ripening showing two waves of activity, the second coinciding with the ripe fruit stage (Sánchez-Sevilla et al., 2021). The role of autophagy in over-ripening fruit remains to be fully elucidated but may have a dual role in nutrient remobilisation and inhibition of senescence progression.

If harvested at an immature stage, strawberry fruit do not continue to ripen normally after harvest (Van de Poel et al., 2014). If harvested green, although their anthocyanin content rises (Ayala-Zavala et al., 2004), metabolite changes do not follow the normal pattern on the plant and aroma development is impaired (Van de Poel et al., 2014). Strawberry fruit are therefore typically harvested just before red ripe stage, and rapidly chilled to delay over-ripening. When stored at 0-5°C, shelf life, as assessed by appearance, is approximately 7-10 days, depending on the cultivar (Pelayo et al., 2003; Vicente et al., 2005).

Transcriptomic studies have compared strawberry fruit ripening stages (Sánchez Sevilla et al., 2017; Wang et al., 2017) revealing a down-regulation of genes associated with photosynthesis, changes in sugar metabolism and increase in flavonoid gene expression. Changes were noted in relation to several major phytohormones: notably GA and IAA associated genes appeared to be down-regulated in the later stages of ripening. Indeed, exogenous treatment with GA and IAA delays ripening while ABA promotes it (Gu et al., 2019). Oxidative phosphorylation was also down-regulated as the fruit reddened (Wang et al., 2017) and it was suggested that this may be an important regulatory step. Postharvest storage at 10°C for up to 7 days (Min et al., 2020) identified groups of transcription factors that were either down- or up-regulated including WRKY, NAC MYB and ERF families. Treatment with phytohormones postharvest (Chen et al., 2016), oF fruit stored at ambient temperature for 2 days showed that auxin delayed postharvest ripening whereas ABA accelerated it. A study of changes in polyphenol content during fruit storage at 0°C over a 10 day period (Koyama et al., 2022) identified changes in expression of genes for several key enzymes in the phenylpropanoid biosynthesis pathway which may be linked to changes in polyphenol content in response to cold storage.

Strawberries are highly valued for their aroma; however, this deteriorates during postharvest storage, and the shelf-life based on maintenance of aroma can be several days shorter than shelf-life based on appearance (Pelayo et al., 2003). Strawberry aroma is composed of a complex mixture of VOCs comprising a heterogeneous group of alcohols, aldehydes, esters, furans, ketones, lactones, organic acids, sulphur compounds and terpenes. Over 900 VOCs have been reported in strawberry volatilomes (Ulrich et al., 2018), however there appears to be high variability across experiments due in part to varietal differences but also methods of analysis. Methyl and ethyl esters of butanoic and hexanoic acids are highly represented in the strawberry volatilome, although there is variation across studies. A total of 36 VOCs were identified as odour active in more than one report (Ulrich et al., 2018), with ethyl butyrate reported in seven independent studies and butyric acid, ethylhaxanoate, DMMF, γ-decalactone and linalool reported in six out of seven studies included (Ulrich et al., 2018). However, there is considerable variability across cultivars, with the relative abundance of the VOC components in the bouquet producing the characteristic aroma. Strawberry fruit VOCs have a dual role in attracting frugivores and in defence; wounding elicits the production of new VOCs from strawberry fruit such as trans-2-hexenal which has antimicrobial properties (Myung et al., 2006).

Postharvest storage impacts the overall VOC profile negatively. As well as a reduction in the production of volatile compounds there is an increase in their breakdown (Kim et al., 2013). For some applications such as inclusion in ready-to-eat fruit salads, strawberries are halved or sliced. As with other fruit such as melons, this increases the surface area for dehydration, affecting VOC profiles (Spadafora et al., 2019). In strawberries, the loss of compartmentation of enzyme and substrates, creates a transient increase in VOCs responsible for an intense fresh-cut aroma. These VOCs include C6 compounds including alcohols, aldehydes and esters from the lipoxygenase pathway and are emitted rapidly but their relative abundance falls off in the 3 h following wounding (Hamilton-Kemp et al., 2003). However, damage to the fruit accelerates decay and microbial colonisation, causing a loss of aroma and flavour for the consumer. Unpleasant-smelling VOCs such as ammonia, high concentrations of ethyl acetate (Forney et al., 2000), and simple alcohols, aldehydes and ketones such as ethanol, acetaldehyde and acetone accumulate due to anaerobic respiration (Kim et al., 2013).

Esters are the major component of the strawberry VOC profile (Jetti et al., 2007), comprising up to 90% of the VOCs, representing over 130 different compounds, and contributing to the fruity notes of the aroma. The final stage of their biosynthesis requires an alcohol acyltransferase (AAT), which catalyzes the esterification of acyl groups from acyl-CoA onto alcohols (Forney et al., 2000) producing butyl, hexyl and octyl acetates. Strawberry AAT is encoded by at least two genes *SAAT* and *FaAAT2* (Parra-Palma et al., 2019) both of which increase in expression through strawberry fruit ripening in parallel with enzyme activity. Expression of SAAT enzyme *in vitro* has shown that it lacks specificity and that the esters generated are largely dependent on the alcohol substrates available (Beekwilder et al., 2004), while FaAAT2 showed activity against C1-C8 straight chain alcohols as well as aromatic alcohols with a preference for cinnamyl alcohol (Cumplido-Laso et al., 2012). However, AAT specificity or substrate preference may vary across cultivars (Olías et al., 2002).

Here VOC and transcriptome analysis of halved strawberry fruit stored at 8°C are used to identify the major changes in aroma, and pathways that are altered during storage of processed fruit suitable for ready to eat salads. Co-regulated transcription factors with AAT suggest potential upstream regulators for this important gene during storage.

## 2 Materials and methods

### 2.1 Plant material and postharvest treatments

Strawberry fruit (*Fragaria x ananassa* cv. Elsanta) were field grown near Cardiff (UK) by the same commercial grower in 2014, 2016 and 2017, transplanted into the field in May and collected at the same point in the growth season (end of July; Dhorajiwala et al., 2021). Weather data are presented in **Supplementary Figure 1**. Fruit were harvested by hand at ripe red stage (Jia et al., 2011) and processed within 3 hours. Fruit were selected for uniformity of size (approximately 2 x 5 cm) and lack of external deformities or damage. They were washed with water, the calyx was removed using a sharp blade, and they were immersed in 200 ppm sodium hypochlorite for 2 min. They were then air dried in a laminar air flow hood and halved. In 2014 and 2016 the two halves were separated. One half of each fruit was stored at 4°C and the other half at 8°C. Fruits were sampled before storage and then at four further time points in 2014 (1, 5, 7 and 12 days), while in 2016 the 7-day time point was omitted. In 2017 fruit was only stored at 8°C and sampled for RNA extraction at 0, 1, and 5 days of storage. Samples for RNA and metabolite analysis were rapidly sliced and snap frozen in liquid nitrogen then stored at -80°C until used. Three replicates composed of 12 half-fruits per replicate were used at each sampling time.

### 2.2 VOC collection and analysis

VOC collection and analysis was performed as described in Amaro et al. (2018) with minor modifications. Samples of halved fruit were placed in a sealed 25 cm x 38 cm nalophene plastic bag (TJM Ltd) and equilibrated for 2 h at room temperature (20°C). A control bag without fruit was included. Headspace (400 mL) was collected using a hand pump (Easy VOC pump, Markes International Ltd.) onto thermal desorption tubes packed with Tenax TA and SulfiCarb sorbents (Markes International Ltd.). A TD100 (Markes International Ltd.) was used to desorb tubes at 120°C for 5 min under a nitrogen flow of 40 mL/min then 260°C for 5 min and recollected to the trap at 24°C. The trap was desorbed from 300°C (heating rate 24°C/s) with a trap hold of 5 min. The split flow rate used was 40 mL/min with a split ratio of 11:1 into the GC (7890A, Agilent Technologies, Inc.). VOCs were separated on a 60 m length, 0.32 mm I.D. and 0.5 µm film thickness Rxi-5ms (Restek) capillary column using the temperature programme: 40°C for 5 min, 10°C/min ramp to 300°C, and a final hold of 5 min (total run time 41 mins). A BenchTOF-dx MS (Almsco International) was used to detect VOCs with source temperature of 275°C, filament voltage 1.6V and m/z range of 35-500m/z. A retention time standard (C8-C20, Sigma Aldrich) was run with each set of samples and prepared by injecting 1μl of the standard mixture directly onto a collection tube (Tenax TA).

VOC data were processed and analysed using AMDIS (NIST 2014) and MSD ChemStation software (E.02.01.1177, Agilent Technologies, Inc.). A custom MS library was produced using retention indices: MS spectra were searched against the NIST library and putative identification was made on basis of >80% match of mass spectra in forward and backward fit and ± 15 in retention index to the custom library. Compounds not present in at least two replicates of one sample were removed, as well as compounds present at similar levels in control samples and VOCs known to be contaminants from plastics and other process-related sources. The clean data underwent normalization using the grand total area abundance and a square root transformation to reduce the effect of highly abundant compounds. Thereafter VOC relative abundance was used as the unit of analysis.

Data were analysed using R software (R i386 2.15.3). PerMANOVA (Permutational Multivariate Analysis of Variance) and CAP analysis (Canonical Analysis of Principal coordinates) statistical tests were performed to identify significant differences amongst whole VOC profiles of the samples over time and at the two different temperatures using ‘adonis’ from the R package ‘vegan’ and ‘CAP-discrim’ from the R package ‘BiodiversityR’. PerMANOVA directly compares the profiles using the volatiles as a dependent and time and temperature stored at as independent variables. CAP was then used as a pairwise comparison since PerMANOVA does not have a *post-hoc* statistical test. CAP was visualised by means of linear discrimination plots, using the first and second linear discriminant as axes (LD1 and LD2). RandomForest™ (RF) was implemented using the R package ‘randomForest’ and used to identify components in the VOC profile that were critical for discrimination. Mean decrease accuracy is an outcome of RF, showing how important each VOC is in classifying the data reported. The top discriminatory VOCs are presented in descending importance.

### 2.3 RNA extraction

Fruit tissue from each replicate of each sampling point was ground under liquid nitrogen to a fine powder and 150 mg were used for extraction essentially as described in Greco et al. (2014). Briefly, extraction buffer containing 200 mM Tris-HCL pH 8, 1.4 M NaCl, 20 mM EDTA, 2% CTAB and 2% mercaptoethanol (500 µL) was added to the powdered plant material and incubated for 30 min at 60°C. Nucleic acids were extracted twice using chloroform: isoamyl alcohol 24:1 (300 µL), and then polysaccharides were precipitated using ¼ volume potassium acetate 3M pH 4.8, and 1/9 volume chilled 100% ethanol. Following centrifugation at 13000 rpm at 4°C for 20 min in an Eppendorf 5417R refrigerated centrifuge, nucleic acids were precipitated overnight at -80°C with 1/10 volume 3M sodium acetate pH 5.2 and 0.8 volumes of chilled 100% ethanol and recovered by centrifugation for 30 min at 7000 rpm as above at 4°C. Following a 75% ethanol wash, pellets were dried and resuspended in 30μL sterile distilled water.

### 2.4 Transcriptomic analysis

RNA used for transcriptomic analysis was harvested and treated in 2014 (one biological replicate) and 2016 (two biological replicates) to capture seasonal variability within the experiment. RNA integrity was checked on agarose gels and then integrity and purity were assessed using an Agilent 2100 bioanalyzer-RNA 6000 Nano Chip (Agilent Technologies) and using a Qubit fluorometer. Random primed cDNA libraries were prepared using a TruSeq RNA Sample Prep kit (Illumina) and Illumina sequencing was performed using paired-end mode on an Illumina Next Seq 5000 Platform.

Reads were assessed using FASTQ, and Trimmomatic was used to remove all adaptor sequences, as well as leading and trailing low quality or N bases. Over 21 M reads were obtained for each sample. The *Fragaria vesca* Whole Genome v2.0.a1 was used as the reference genome (sourced from Rosecae.org 2017) and the *F. ananassa* sequences were mapped using STAR (Dobin et al., 2012). Sequencing data have been deposited in the NCBI Sequence Read Archive (http://www.ncbi.nlm.nih.gov/Traces/sra) under SRP study accession number PRJNA931751. A feature count of the number of reads mapping to each gene was taken to produce data that could be analysed in R (v 3.4.1) using the DEseq2 module (Love et al., 2014). A model accounting for seasonality (as the RNA samples were derived from fruit collected in two seasons: 2014 and 2016 as well as storage time, was produced. This generated three files of pair-wise comparisons amongst all time points, expressing the count data and relative expression (in the form of Log 2 Fold Change, Log2FC, values) of Differentially Expressed Genes (DEGs). Only significantly differentially expressed genes (Padj < 0.05) corrected using the Benjamini-Hochberg correction were used for further analysis. FASTA files of the sequences for the significant DEGs were produced, allowing for pathway analysis. BLASTx was used to interrogate the nr BLASTdb (BLAST database) to identify gene functions. An alignment cut off e-value of < 1 x 10^-3^ was used. Significantly changed metabolic pathways were identified and functional classification of enriched Gene Ontology (GO) terms were sorted by “Process” using Plant MetGenMAP (Joung et al., 2009). Plant Transcriptional Regulatory Map (PlantRegMap, Jin et al., 2017) was used to analyse significantly up-regulated or down-regulated genes from the RNA-seq data. AgriGo (V2.0, Tian et al., 2017) was used to generate heatmaps from the PlantRegMap GO annotation. Kyoto Encyclopaedia of Genes and Genomes (KEGG) gene IDs were assigned to RNA-seq genes by converting their NCBI refseq accession to KEGG IDs using bioDBnet (Mudunuri et al., 2009; bioDBnet, https://biodbnet-abcc.ncifcrf.gov/db/db2db.php). Pathways were then visualised using KEGG Mapper (Kanehisa and Goto, 2000; https://www.kegg.jp/kegg/tool/conv_id.html) with colours of expression being assigned based on Log2FC of significant DEGs.

Genesis software (Sturn et al., 2002) was used to visualise the significantly (Padj < 0.05) up-regulated and down-regulated genes on day 0, 1 and 5 of storage at 8 ˚C. The software normalises the data by taking the standard deviation for each sample. To cluster the genes, the k-means clustering algorithm was used within the Genesis 1.8.1 software with seven clusters specified. The clustering process was repeated so that those that showed tightest expression to the AAT gene were grouped. The genes that were down-regulated similarly to AAT were compared against the NCBI database by a command line BLAST to obtain functional annotations.

### 2.5 Real time PCR

Extracted RNA was treated with DNase (Promega) and removal of genomic DNA was verified using PUV primers (**Supplementary Table 1**). cDNA was synthesized from 1-2 μg of total RNA using M-MLV reverse transcriptase (Promega), and products again verified with PUV primers.

RNA for real time PCR was extracted from three biological replicates of fruit material harvested and treated in 2017. Real time PCR was performed in 20μL containing 10μL of SYBR Green (2x, PCR Biosystems), 0.4μL of each sequence-specific primer, 6μL of sample cDNA (10μM). Each reaction was carried out in technical duplicates for each cDNA sample and gene. The PCR settings used were: 95°C for 2 min, 35 cycles of 95°C 30 s, 55°C 30 s, 72°C 30 s, then 95°C for 30 s, 55°C for 60 s, 97°C for 1 s. Only Ct values with a range of 0.2 were used to normalise data and calculate relative expression.

Primers were designed using Primer3 Software (http://primer3.ut.ee/) (Rozen and Skaletsky, 2000) following alignment of the RNAseq reads to the *F. vesca* genome sequence, in Integrated Genome Browser (IGB 9.0.2; Freese et al., 2016), selecting regions where base differences between the *F. vesca* and *F. ananassa* genes would not affect the primer efficiency. Primers were designed at the 3’ end of the selected genes and designed to yield products of approximately 150-200 bp (All primers are listed in **Supplementary Table 1**).

### 2.6 Analysis of epicatechin content

Extraction and hydrolysis of phenolic compounds was adapted from (Tarola et al., 2013). A sample of 0.25 g of frozen strawberry fruit material was mixed with 0.5 mL of HPLC-grade methanol and sonicated for 15 min at room temperature. The mixture was centrifuged at 3000 rpm (Z606235 SIGMA Eppendorf^®^ Minispin^®^ personal microcentrifuge) for 15 min and the supernatant collected. Extractions were repeated once again and supernatants combined. Extracts were dried under a flow of nitrogen and resuspended in 0.5 mL methanol (HPLC grade, Fisher). Samples were filtered through a 0.45 µm pore-size filter (Millipore) before injection into the HPLC. HPLC analyses were carried out on a ThermoScientific HPLC system consisting of P4000 quaternary pump, AS300 autosampler and photodiode array detector (UV6000LP). Samples (20μL) were analysed over a reversed phase C18 column at a flow rate of 1 mL/min and using the following gradient program: 0.01–8.00 min 17% B, 8.01–25.00 min 17–59% B, 25.01–40.0min 56% B, 40.01–50.0 min 56–100% B, and 50.01–55.00 min 100–17% B. Solvent A was 2% formic acid in H_2_O and solvent B 2% formic acid in 90% acetonitrile (Tarola et al., 2013). A stock solution of standard (10 mmol/L) was prepared by dissolving catechin (≥ 95%), in methanol (HPLC grade, Fisher). Calibration standards for quantitation (5-60 mg/l) (Tarola et al., 2013) in light-protective glassware and stored at -20°C until used.

## 3 Results

### 3.1 VOC profiles discriminate storage time but abundance varied between the two seasons

**Figure 1:**
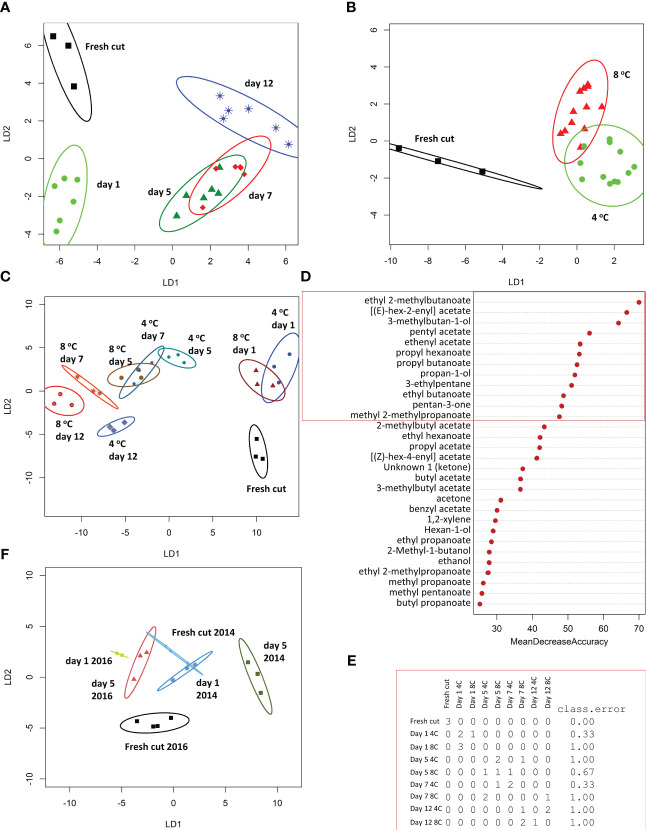
Analysis of strawberry fruit VOCs during storage: CAP analysis of VOCs from 2014 season fruit stored for up to 12 days at 4°C or 8°C: **(A)** time of storage **(B)** temperature of storage **(C)** combined time (0, 5, 7, and 12 d) temperature. **(F)** VOCs shared between 2014 and 2016 season strawberries stored at 8°C for 5 days. Each ellipse represents the 95% confidence interval. The plots use LD1 and LD2 with a percentage of correct classification of **(A)** 63% [n=3-6]); **(B)** 85% (n=3-12), **(C)** 48% (n=3) and **(F)** 83% (n= 2-4). **(D, E)** Random Forest analysis of 2014 VOCs **(D)** Mean decrease accuracy (12 most discriminatory VOCs are boxed); **(E)** confusion matrix showing classification errors for each sample.

In the 2014 season 63 VOCs were detected across all samples representing 9 families: 30 non acetate esters, 15 acetate esters, 6 alcohols, 6 ketones, 2 sulphur containing VOCs, and one each of acids, alkanes, aromatic, and furan VOCs (**Supplementary Table 2**). At all time points and temperatures, esters were the most abundant family (**Table 1A**). The VOC profiles were significantly different across time points of storage, based on PerMANOVA (P < 0.001), but not with respect to temperature of storage. Based on linear discrimination (LD) plots produced by CAP, the whole VOC profile from the 2014 season discriminated clearly between fresh cut (day 0), day 1 of storage, and later storage time points (**Figure 1A**). While storage for 5 days was discriminated from storage for 12 days, the VOC profile at day 7 overlapped both day 5 and day 12 of storage. VOC profiles of all samples of strawberries stored at 8°C or 4°C were not discriminated in CAP plots, however stored samples were discriminated from fresh cut (**Figure 1B**). When VOC profiles from individual samples were analysed using CAP, discrimination between the two temperatures of storage was possible after day 1, but the VOC profile from strawberries stored at 4°C for 7 days overlapped the profiles of strawberries stored at either temperature for 5 days (**Figure 1C**). Random Forest analysis was used to identify the VOCs that most contribute to the discrimination across time and temperatures (**Figures 1D, E**). The confusion matrix indicates that discrimination was best between fresh cut fruit and all the stored fruit (classification error of 0), as also shown with the CAP analysis, but VOCs on day 1 were discriminated from other time points, and day 7 VOCs from fruit stored at 4°C were discriminated from other VOC profiles apart from fruit stored at 8°C for 5 days. Other timepoints were less well discriminated. Eight of the 12 most discriminatory VOCs (**Figure 1D**) were esters, three of them acetate and the rest non-acetate.

**Table 1:** total mean % abundance of VOC families in (A) 2014 and (B) 2016.

(A) 2014		4°C	8°C	4°C	8°C	4°C	8°C	4°C	8°C
family	Day 0	Day 1		Day 5		Day 7		Day 12	
**acids**	0.27a	0.00a	0.00a	0.70a	0.67a	0.47a	0.25a	0.21a	0.02a
**alcohol**	11.7b	4.56a	4.05ab	6.47ab	0.53a	4.61a	0.76a	19.6b	6.58ab
**alkane**	0.55a	0.20a	0.31a	0.00a	0.00a	0.00a	0.00a	0.00a	0.00a
**aromatic**	0.02a	0.01a	0.01a	0.00a	0.00a	0.00a	0.01a	0.00a	0.00a
**esters (acetate)**	43.0d	30.2b	39.6c	29.1bc	54.0c	39.18a	55.4b	56.4c	45.2b
**esters (non-acetate)**	35.0c	56.0c	48.8d	44.5c	21.2b	15.0a	31.4ab	8.83ab	37.8ab
**furan**	0.00a	0.00a	0.00a	0.01a	0.01a	0.00a	0.00a	0.01a	0.01a
**ketone**	9.47b	8.94a	7.16b	19.0ab	23.4b	40.6a	12.1a	15.3ab	10.3ab
**sulphur**	0.02a	0.04a	0.13a	0.14a	0.20a	0.21a	0.11a	0.10a	0.01a
(B) 2016		4^o^C	8^o^C	4^o^C	8^o^C	4^o^C	8^o^C		
family	Day 0	Day 1		Day 5		Day 12			
**acid**	1.77a	2.60a	1.64a	3.41a	2.63a	0.45a	0.45a		
**alcohol**	0.09a	0.22a	2.47a	0.14a	1.88a	0.32a	0.32a		
**aldehyde**	0.00a	0.00a	0.00a	0.00a	0.00a	0.34a	0.34a		
**alkane**	0.00a	0.00a	0.00a	0.00a	0.00a	0.07a	0.07a		
**carbamate**	0.10a	0.22a	0.23a	0.17a	0.15a	0.08a	0.08a		
**epoxy**	0.00a	0.00a	0.02a	0.00a	0.03a	0.00a	0.00a		
**esters (acetate)**	14.2a	28.4b	28.8b	30.9b	28.1b	20.3ab	20.3ab		
**esters (non-acetate)**	50.1a	67.9c	64.9c	65.1c	66.4c	45.0b	45.0ab		
**sulphur**	20.4a	0.66a	1.92a	0.29a	0.77a	0.01a	0.01b		
**terpene**	0.32a	0.08a	0.09a	0.04a	0.02a	0.16a	0.16a		

Different letters indicate significant differences in relative abundance amongst families for each treatment/timepoint.

To assess how reproducible the VOC profiles were across seasons, strawberry VOCs were assessed also in 2016 across 12 days of storage at 8°C. Across all samples from both years, 90 VOCs were detected, representing 12 families: non-acetate esters (43), acetate esters (17), alcohols (8), ketones (6), sulphur containing (4), alkanes (3), two aldehydes and acids, and a single VOC of aromatic, epoxy, carbamate, furan and terpene families (**Supplementary Table 2**). Again, esters were the most abundant family at each timepoint and treatment (**Table 1B**). Similar numbers of VOCs were found in the two years (63 in 2014 and 60 in 2016), but only 33 were shared between the two years. PerMANOVA analysis of the 2016 VOC dataset indicated significant differences amongst the days of storage (P = 0.030). However, discrimination by temperature of storage was not statistically significant. Moreover, based on linear discrimination plots produced by CAP, the whole VOC profile from the 2016 season was not able to discriminate between either days or temperatures of storage (**Supplementary Figure 2**).

The relative abundance of the 33 VOCs common to the two seasons were assessed using PerMANOVA to determine whether they might be better discriminators. This analysis was restricted to the 8°C storage temperature and the 0, 1 and 5 days storage time points as these were of relevance to the transcriptomic analysis and also more relevant to retail storage conditions and time of storage. Analysis using PerMANOVA did not indicate significant discrimination across all samples based on day of storage. However, linear discrimination plots derived from CAP showed good discrimination for the 2016 VOCs across the three storage time points, although this shared reduced dataset was unable to discriminate between fresh cut and fruit stored for 1 day at 8°C from the 2014 dataset, and moreover the fresh cut sample VOC profile from 2014 overlapped with the day 5 sample from 2016 (**Figure 1F**).

### 3.2 Global transcriptomic changes during postharvest storage

Transcriptomic analysis was used to assess global changes in transcription across three storage timepoints in halved strawberry fruit: day 0, 1 and 5 at 8°C. The aim was to identify key pathways affected by cold storage and potential regulators for expression changes, in particular for changes to the AAT expression that may be contributing to changes in the VOC profile.

**Figure 2:**
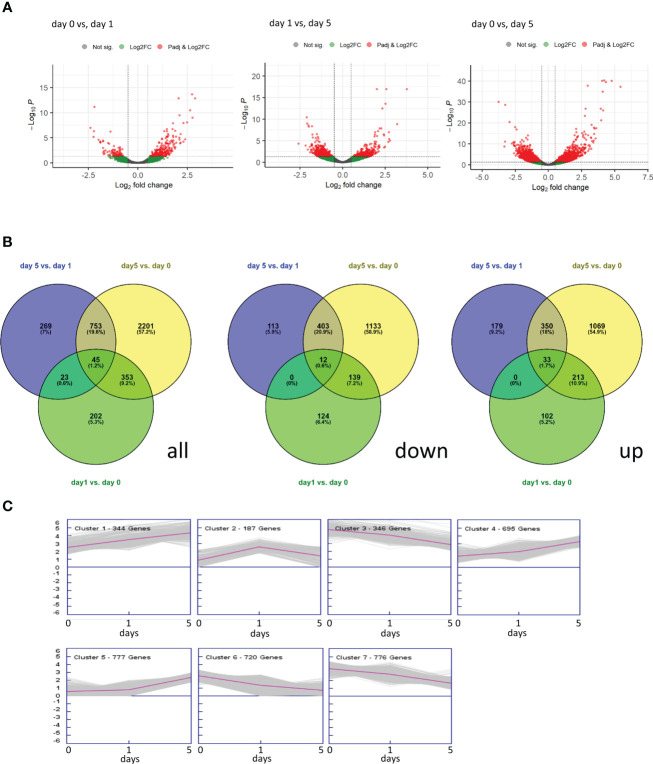
Overview of Differentially Expressed Genes (DEGs, p adj. < 0.05) across the three sampling times (0, 1 and 5 days) of halved strawberry fruit stored at 8°C. **(A)** Volcano plots derived from DESeq2 (red dots indicate DEGs), **(B)** Venn diagrams of all, down-regulated and up-regulated DEGs, **(C)** Clustering of the DEGs into seven expression profiles.

Between 20.7 M and 53.6 M reads were obtained for each of the samples (**Supplementary Table 3**) of which 89% were mapped onto the *F. vesca* genome resulting in 24,724 mapped genes. A PCA of the mapped reads grouped the two replicates from 2016 closely, but there was substantial variability across the two harvest years (**Supplementary Figure 3**). Nevertheless, PC2 separated the day 0 and day 1 replicates clearly from day 5 replicates with some separation also between day 0 and day 1. Volcano plots (**Figure 2A**) indicated that there were most DEGs between samples at day 0 and day 5 of storage and that more DEGs were up rather than down regulated between day 0 and day 1. Of a total of 3846 DEGs (p adj. < 0.05), indeed the majority changed between day 5 and day 0 of storage (3352) with 57.2% (2201) unique to this comparison (**Figure 2B**). Similar numbers of genes were down- (1924) and up-regulated (1946) and the proportions shared between the different comparisons were also similar.

The DEGs were clustered into seven co-expression groups (**Figure 2C**). The largest cluster (cluster 5) contained genes with a generally upward trend in expression, while the next two largest clusters (clusters 7 and 6, containing 776 and 720 genes respectively) showed a general downward expression trend between 0 and 5 days of storage. Only one cluster, cluster 2 contained genes that were up-regulated between day 0 and day 1 but then down-regulated between day 1 and day 5.

### 3.3 Functional analysis of gene expression changes

**Figure 3:**
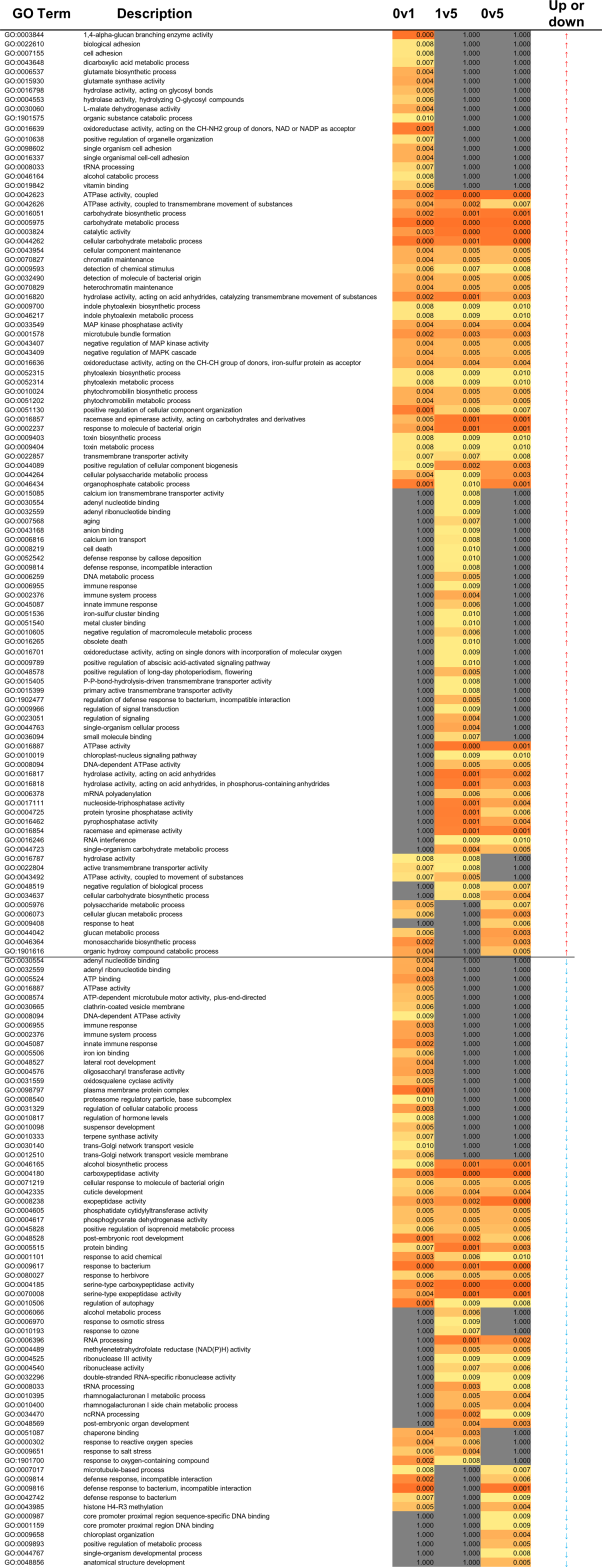
Heatmap of molecular function for strawberry GO enrichment terms. 0, 1 and 5 relate to days of storage. Darker orange indicates more highly significant terms (p < 0.05). Grey indicates that the GO term was not enriched in that comparison (p = 1). The last column indicates whether the term was derived from transcripts that were significantly up- or down-regulated.

GO term analysis was used to analyse global changes in expression across the three time points of strawberry fruit storage. This was used to assess whether changes were progressive or were limited to earlier (day 0 to day 1) or later (day 1 and day 5) time points (**Figure 3**). There were more up- (99) than down- (66) regulated GO terms and all the GO terms were consistently up- or down-regulated across the different time point comparisons. Of the up-regulated GO terms, 29 were up-regulated at all time comparisons and included processes such as ATPase activity, carbohydrate metabolism, processes related to MAP kinases and phytoalexins. Processes only up-regulated between day 0 and day 1 (14 GO terms) included glutamate biosynthesis, and hydrolase activities. In contrast processes only up-regulated between day 1 and day 5 (27 GO terms) include ageing, cell death, defence and immune responses, ABA and other signalling processes. The down-regulated GO terms associated with genes down-regulated in all comparisons (15 GO terms) included peptidase activities, alcohol biosynthesis, cuticle development and several terms linked to responses to biotic and abiotic agents. Twenty-two early (day 0 to day 1) GO terms linked to down-regulated genes included immune responses, developmental processes, terpene synthase, and Golgi transport. Only three GO terms were associated only with later down-regulated genes (day 1 to day 5) and these related to responses to abiotic stress and alcohol metabolism.

### 3.4 Enriched metabolic pathways

Plant MetGenMap was used to identify pathways that were significantly enriched in the different sets of DEGs (**Table 2**). Some pathway enrichment was specific to the time interval: for example, sucrose degradation was only enriched in the day 0 vs. day 1 comparison, and seven pathways were specific to the day 1 vs day 5 comparison. These included farnesene and linalool biosynthesis both involved in VOC biosynthesis. Nitrogen metabolism and cellulose biosynthesis were enriched in the day 0 vs. day 5 comparison and starch biosynthesis was enriched in both day 0 vs. day 1 and day1 vs day 5 while serine biosynthesis enrichment was shared in the day 0 vs. day 1 and day 0 vs. day 5 comparisons.

**Table 2:** Significantly changed pathways based on transcriptomic data for strawberry post-harvest storage (based on analysis in Plant MetGenMap.

Comparison	Pathway	p value
Day 0 vs. Day 1	Guanosine nucleotides *de novo* biosynthesis	0.007
	Starch biosynthesis	0.022
	Serine biosynthesis	0.029
	Sucrose degradation (III)	0.049
Day 1 vs. Day 5	Farnesene biosynthesis	<0.001
	Linalool biosynthesis	0.002
	Glycogen biosynthesis (from ADP-D-glucose)	0.017
	Ubiquinone-10 biosynthesis (eukaryotic)	0.035
	δ-carotene biosynthesis	0.035
	Ascorbate biosynthesis II (L-gulose pathway)	0.035
	Pentose phosphate pathway	0.042
	Starch biosynthesis	0.049
Day 0 vs. Day 5	Tryptophan biosynthesis	<0.001
	Glutamate biosynthesis II	0.013
	Glutamine degradation II	0.013
	Glutamate biosynthesis I	0.013
	Cellulose biosynthesis	0.025
	Serine biosynthesis	0.036
	2-ketoglutarate dehydrogenase complex	0.036

**Figure 4:**
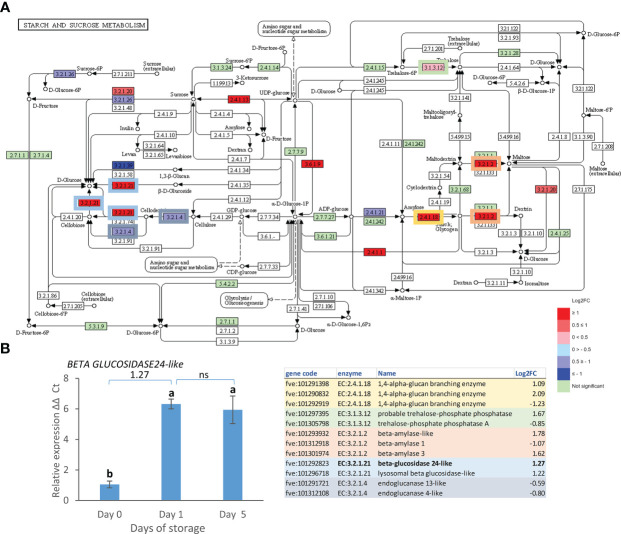
Day 0 vs. day 5 DEGs in the starch and sucrose metabolism pathway. **(A)** Pathway adapted from KEGG (Fve). Significantly up and down regulated enzymes are shown in red and blue respectively. Green coloured enzymes are encoded by strawberry genes which did not change significantly in this transcriptome comparison. Enzymes which are encoded by multiple genes in the transcriptome are boxed, and box outline colour indicates the respective shaded section in the table. **(B)** Expression of *BETA GLUCOSIDASE24-like* gene over 5 days post-harvest (8°C). Means ± SE; letters correspond to significant differences (*p* =< 0.05) by one-way ANOVA. Log2 FC in transcriptome data is shown above the real time PCR data. ns, non significant.

A parallel analysis in KEGG highlighted 77 pathways in day 0 vs. day 1, 88 in day 1 vs. day 5 and 121 in day 0 vs. day 5. Of these, starch and sugar metabolism, and phenylpropanoid biosynthesis pathways included most gene expression changes in the day 0 vs. day 5 comparison while the autophagy pathway showed more changes in the day 1 vs. day 5 comparison. Starch and sugar metabolism showed an up-regulation of genes leading to production of glucose, sucrose, dextrin, maltose and trehalose (**Figure 4A**) suggesting breakdown to simple sugars. The up-regulation of expression of the strawberry genes encoding a beta-glucosidase 24-like enzyme (EC:3.2.1.21) responsible for the conversion of cellobiose, cellodextrin and fructose to glucose during postharvest storage was confirmed by real time PCR (**Figure 4B**).

**Figure 5:**
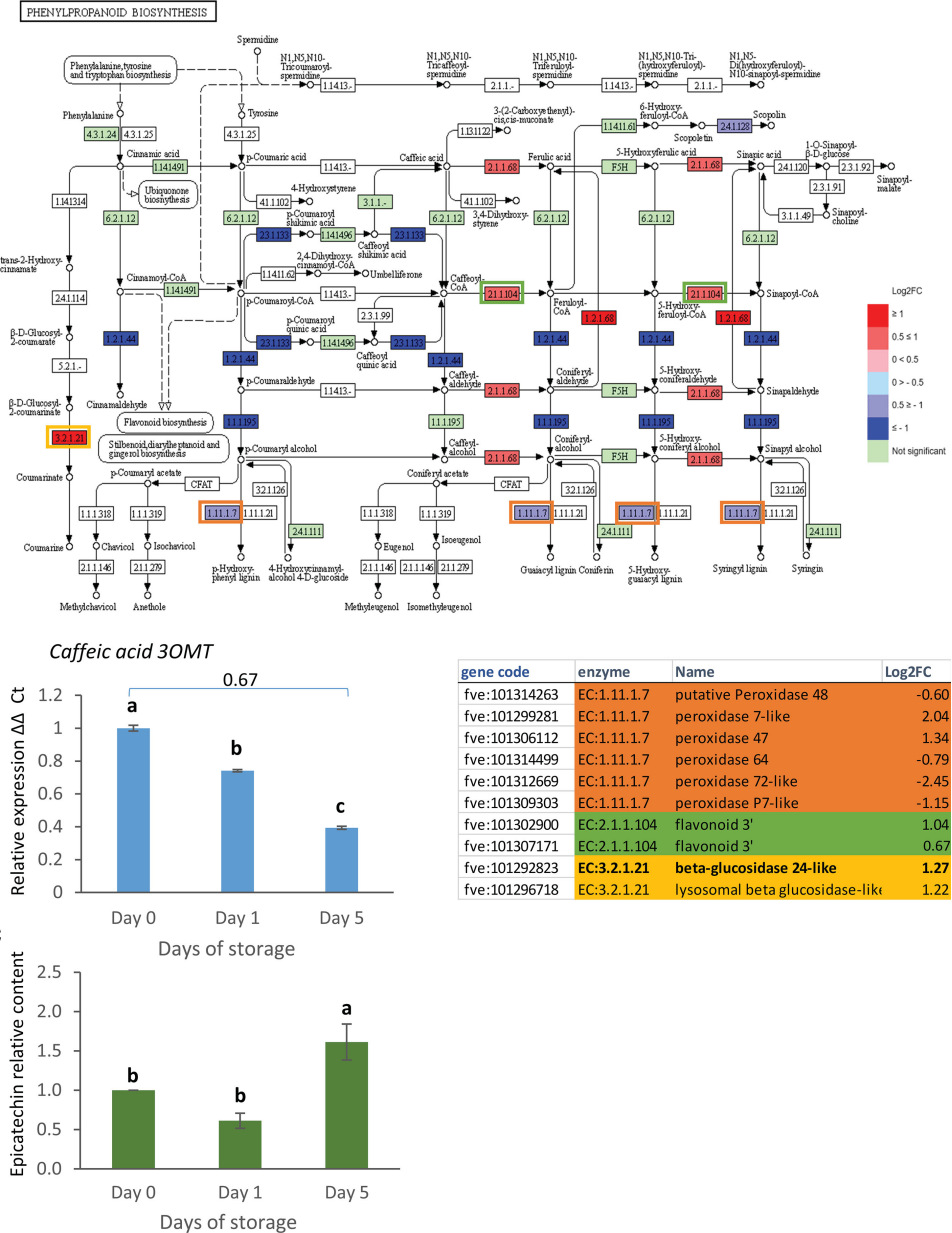
**(A)** Day 0 vs. day 5 DEGs in the phenylpropanoid biosynthesis pathway. Pathway adapted from KEGG (Fve). Significantly up and down regulated enzymes are shown in red and blue respectively. Green coloured enzymes are encoded by strawberry genes which did not change significantly in this transcriptome comparison. Enzymes which are encoded by multiple genes in the transcriptome are boxed, and box outline colour indicates the respective shaded section in the table. **(B)** Expression of *Caffeic acid 3OMT* and **(C)** change in epicatechin content as assessed by HPLC (µg/g FW) over 5 days post-harvest (8°C). Means ± SE; letters correspond to significant differences (*p* =< 0.05) by one-way ANOVA. Log2 FC in transcriptome data is shown above the real time PCR data.

The phenylpropanoid biosynthesis pathway showed strong down-regulation of EC:1.1.1.195 (cannabidiolic acid synthase-like 1), EC:1.2.1.44 (cinnamoyl-CoA reductase 1-like) and EC:2.3.1.133 (vinorine synthase-like) in the day 0 vs. day 5 comparison (**Figure 5A**). Peroxidases [EC:1.11.1.7] were also slightly down-regulated. These enzymes are involved in the synthesis of lignin-related compounds but also potentially in the production of VOCs related to eugenol. In contrast, aldehyde dehydrogenase [EC:1.2.1.68] leading to the production of ferulic and sinapic acid were strongly up-regulated, as well as the beta glucosidase [EC:3.2.1.21] related to coumarine production. The latter has the same enzyme code as the beta glucosidase in the starch and sucrose metabolism pathway and up-regulated expression of its transcript was already confirmed by real time PCR (**Figure 4B**). Another enzyme related to secondary metabolism, caffeic acid 3OMT [EC:2.1.1.68], related to furanone VOC biosynthesis was weakly up-regulated according to the transcriptome analysis but its transcript decreased in expression when analysed by real time PCR (**Figure 5B**). Most phenolics analysed previously (catechin, rutin and quercetin) did not change in content during postharvest storage at 8°C over a 5 day period (Dhorajiwala et al., 2021). However, epicatechin levels remained stable for the first day of storage but then increased significantly after 5 days of storage (**Figure 5C**).

**Figure 6:**
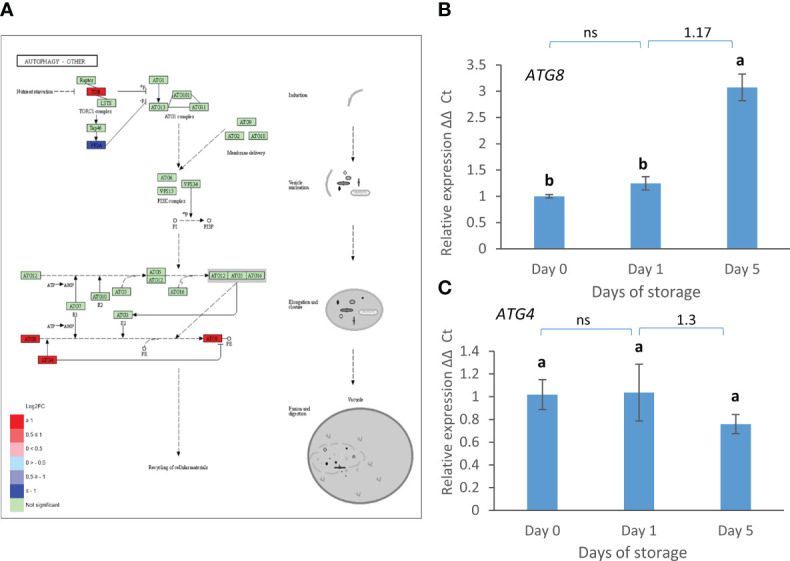
D1 vs. D5 DEGs in the autophagy pathway. **(A)** Pathway adapted from KEGG (Fve). Significantly up and down regulated enzymes are shown in red and blue respectively. Green coloured enzymes are encoded by strawberry genes which did not change significantly in this transcriptome comparison. **(B)** Expression of ATG8 and **(C)** ATG4 over 5 days post-harvest (8°C). Means + SE; letters correspond to significant differences (p =< 0.05) by one-way ANOVA. Log2 FC in transcriptome data is shown above the real time PCR data. ns, non significant.

Genes encoding three components of the autophagy pathway: *TOR*, *ATG8* and *ATG4* were strongly up-regulated between day 1 and day 5 (**Figure 6A**). The strong up-regulation of *ATG8* but not *ATG4* was confirmed by real time PCR (**Figures 6B, C**).

### 3.5 Expression of transcription factors during strawberry fruit storage

**Figure 7:**
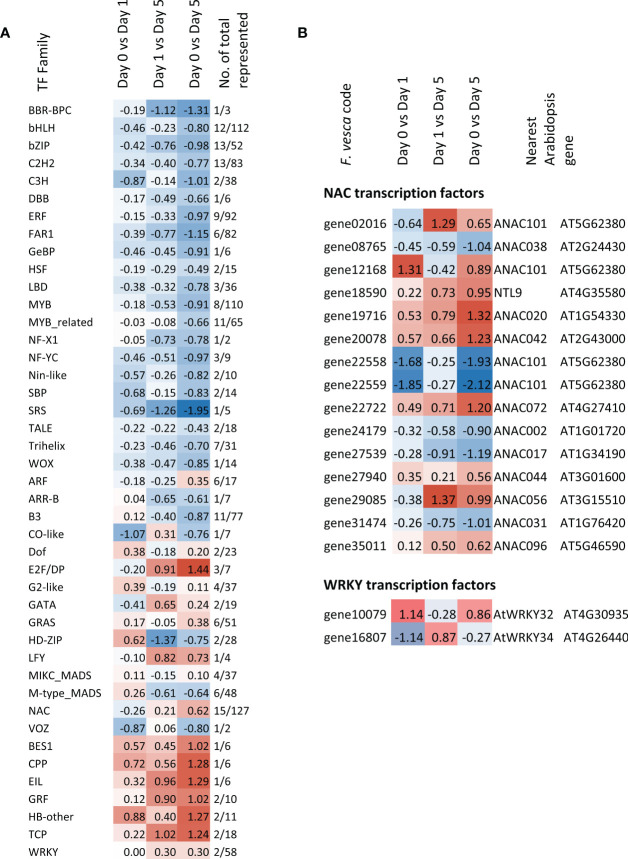
Heatmap of **(A)** median Log2FC expression for TF gene families **(B)** expression of NAC and WRKY family members represented in the strawberry fruit postharvest storage transcriptome over each time comparison. Genes used to generate the median expression were significant in at least one time point comparison. (Morpheus, https://software.broadinstitute.org/morpheus).

Genes belonging to a total of 43 transcription factor (TF) families were represented amongst the DEGs (**Figure 7A** and **Supplementary Table 4**). Of these, the median expression of seven was up-regulated across all three storage time comparisons and were the BES1, CPP, EIL, GRF, HB-other, TCP and WRKY families. In contrast, 21 of the TF families represented genes that were consistently down-regulated. These included the bHLH, bZIP, C2H2, ERF, FAR1, HSF, MYB and WOX families amongst others. In each case the up- or down-regulation between days 0 and 5 was greater than between day 0 and day 1. A more detailed examination of two stress-related TF families, WRKY and NAC reveals members that are both up- and down-regulated. Six NAC TFs were significantly down-regulated between day 0 and 5, six NACs showed a consistent up-regulation and six showed a consistent down-regulation across the timepoints (**Figure 7B**). The two WRKY genes showed a mixed pattern of expression with the *WRKY32-like* gene up-regulated after one day of cold storage but down-regulated between day 1 and day 5 and then *WRKY34-like* gene showing an opposite pattern.

### 3.6 Identifying potential regulators of AAT expression

**Figure 8:**
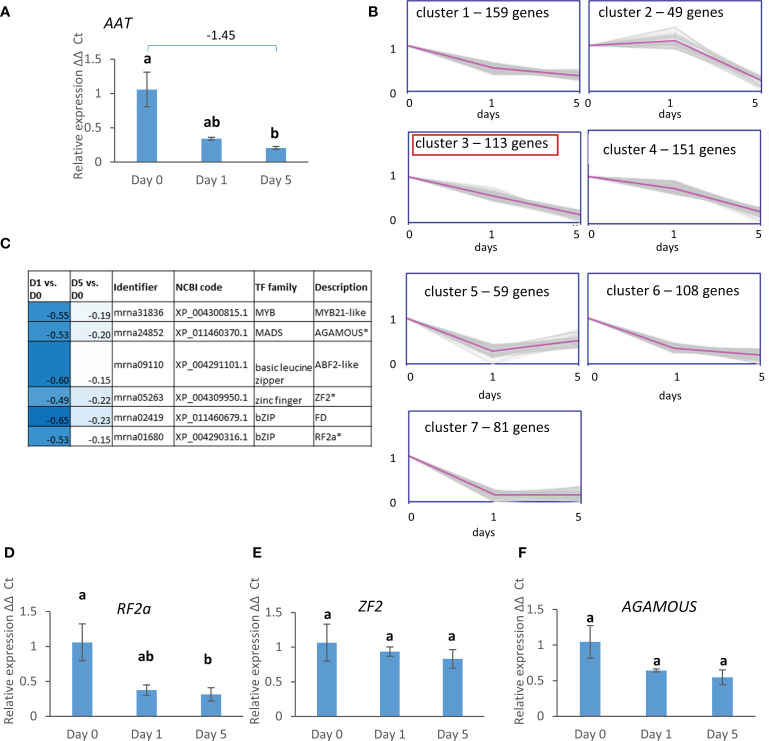
**(A)** Expression of *FaAAT*, **(B)** k-means clustering of the 720 genes with the most similar expression pattern to *FaAAT: FaAAT* is located in cluster 3 together with 112 other genes which share a downwards trend over time of storage. **(C)** heat map of the seven genes within cluster 3 that are annotated as transcription factors, **(D-F)** expression of three TFs shown in **(B)**. Expression data are over 5 days post-harvest (8°C). Means ± SE; letters correspond to significant differences (*p* =< 0.05) by one-way ANOVA.

Since esters were a major component of the VOC profiles, expression of *AAT* was assessed over the same storage period and conditions (**Figure 8A**) showing a significant reduction in expression between day 0 and day 5 of storage at 8°C. k-means clustering was then used to refine the group of DEGs co-expressed with *AAT* with the aim of identifying potential co-expressed regulators. *AAT* was contained in cluster 6 (**Figure 2B**) containing 720 genes. These 720 genes were re-clustered into seven sub-clusters (**Figure 8B**). *AAT* was found in cluster 3 containing 113 genes that were continuously down-regulated from day 0 through to day 5. Six of the genes within this cluster are annotated as transcription factors (**Figure 8C**) including one each of MYB, MADS, basic leucine zipper, and zinc finger protein families and two bZIP family genes. Expression was down-regulated to different extents, but all were more strongly down-regulated after the first day of cold storage than after five days. Real time PCR confirmed the significant down-regulation of *RF2a* (**Figure 8D**). *AGAMOUS* also appeared to be down-regulated with cold storage, but the changes were not significant, and there was only a slight detectable reduction in mean relative expression with storage for *ZF2* (**Figures 8E, F**).

## 4 Discussion

Despite a similar number of VOCs detected in each season, only about half of them were detected in both seasons. Cultivar and maturity stage have a large impact on VOC profiles, but specific growing conditions (climate and cultural practices) may also have an impact as well as post-harvest storage temperature (Forney et al., 2000), and postharvest handling methods. The method of VOC analysis (Ulrich et al., 2018) can also affect the VOC profiles detected. However, it is not always easy to determine which factor has the greatest effect as there are few reports of VOC analysis for the same cultivar at the same stage of ripening and month of collection, stored at the same conditions and analysed in exactly the same way over two seasons. In particular, effects of season and storage on halved fruit, suitable for inclusion in ready to eat fruit salads has not been fully investigated. Environmental effects clearly do affect sensorial perception and phytochemical content (Alvarez-Suarez et al., 2014; Vicente et al., 2014) and data here indicate the extent to which VOC profiles can be affected by differences in pre-harvest environment. Both rainfall and temperature varied across the three years of study in each cultivation month between May when the plants are transplanted to the field and the harvest in July (**Supplementary Figure 1**). Most notably 2014 was the warmest year in June and July but not in May, and rainfall was most variable across years in May and July. However, the field environment is complex, and it is difficult to predict the specific effects on the VOC profiles. It is therefore important in assessing new breeding lines, and in optimising flavour for the consumer to consider the aroma across several growing years when grown in the field.

Interestingly changes in VOC profile during storage were much clearer in the 2014 season compared to the 2016 season fruit. In 2014 six of the 30 VOCs that most discriminated the VOC profiles across different storage conditions were amongst the 30 most common strawberry VOCs (Ulrich et al., 2018). These were ethyl hexanoate, ethyl butanoate, butyl acetate, ethyl 2-methylbutanoate, benzyl acetate and pentyl acetate. One of these, ethyl butanoate, was listed as a character impact VOC across a wide range of studies. Three of the four VOCs identified previously as the most important odorant components of fresh strawberry (Kim et al., 2013): ethyl butyrate, ethyl hexanoate and ethyl 2-methyl butanoate, were also amongst the 30 most discriminatory VOCs in the 2014 fruit, as well as ethanol that was associated by Kim et al. (2013) with the development of off odours in decayed fruit. Hence the aroma changes noted would affect key components of the perceived aroma.

Discrimination of VOC profiles across days of storage differed between the two years even when only including in the analysis the 33 VOCs that were shared between the two seasons and restricting the analysis to fruit stored at 8°C. These 33 VOCs were sufficient to discriminate the 2016 fruit across storage time but not the 2014 fruit. This indicates that in some seasons, aroma may change less during storage, dependent on the development of the whole bouquet in the fresh fruit. Thus, the changes across storage noted here may be important in affecting sensorial perception of the aroma and the changes in the most common strawberry VOCs may be of relevance to other cultivars as well. However, the results emphasise that the effects of storage on aroma need to be assessed across multiple seasons even for the same variety of fruit.

As found previously (Ulrich et al., 2018; Yan et al., 2018), esters dominated the VOC profiles of the strawberry fruit. Of the 30 most frequently identified VOCs in strawberries (Ulrich et al., 2018), nine were identified in both seasons studied, eight of which were esters, while a further four VOCs were only detected in the 2014 strawberry fruit and one more in 2016 season fruit. Forney et al. (2000) found changes in specific esters during storage: ethyl butanoate and ethyl hexanoate levels increased after storage for 5 days at 1°C, while methyl butanoate decreased and methyl hexanoate remained approximately stable. Here, none of these four VOCs changed significantly in relative abundance in the 2016 season fruit during storage at either temperature. However, in the 2014 season fruit ethyl butanoate and ethyl hexanoate decreased over storage at 4°C while methyl butanoate and methyl hexanoate decreased when fruit was stored at 8°C (**Supplementary Figure 4**). This suggests an effect of the cold storage in reducing specific esters. The discrepancies with Forney et al. (2000) may relate to the different temperatures tested here, the different cultivars tested and possibly also that fruit here was halved whereas in the Forney et al. (2000) study it was whole.

AAT is the key enzyme responsible for the production of esters, and its gene expression fell with storage in line with the reduction in some of the most important esters in the VOC profile discussed above. Hence as well as gaining an overall picture of gene expression changes, one focus of the transcriptomic analysis was to understand better the expression of AAT through postharvest storage and co-expressed transcription factors that may be relevant to its regulation. To mitigate the effects of seasonal variation noted in the VOC analysis, the transcriptome included fruit from two different seasons. As with the VOCs, there were clear seasonal differences across the replicates which increases the robustness of the DEGs that were identified as changing in relation to storage. Further verification was included by using material from a third season (2017) for the real time PCR, with results being compared to the transcriptome that was generated from two other seasons (2014 and 2016). For seven out of the eight genes tested and shown here, the pattern of change was in agreement between real time PCR and transcriptome although for three of them changes were not statistically significant in the real time PCR. This proportion of agreement between transcriptome and real time PCR is similar to other studies (e.g. Cavaiuolo et al., 2017).

Overall, the GO term analysis indicates different changes occurring in the first day of storage (early responses) and in the successive 4 days (later responses). The up-regulation of ageing and cell death related GO terms later in storage is consistent with fruit that is entering a senescence phase as has been found in other studies of senescent tissues (e.g. Lear et al., 2022). It is also of interest that ABA related genes were only up-regulated later, perhaps when storage dehydration is acting as a trigger. A peak in ABA content was noted on day 2 of storage at 0°C (Koyama et al., 2022) and would be consistent with the subsequent activation of ABA responsive genes. The down-regulation of abiotic stress related genes later in storage is perhaps surprising although it may reflect a general shutting down of metabolism linked to senescence. The down-regulation of terpene synthase genes may be relevant in the VOC changes, also noted here, although terpenes were a minor component of the VOCs detected (only represented by limonene in the 2016 season fruit). Although its relative abundance did not change significantly during storage, limonene did appear to be at its highest relative abundance at day 0. The down-regulation of alcohol biosynthesis and metabolism during storage may also affect the VOC bouquet both as aroma-relevant compounds but also as substrates for ester production.

The majority of the DEGs found here associated with starch and sugar metabolism increased in expression. Starch and sugar metabolism was also an enriched pathway when strawberry fruit were exposed to 0°C for 10 days (Koyama et al., 2022). Most of the enzymes encoded catalyse the production of monosaccharides: glucose, maltose, and fructose or dextrin, a breakdown product of starch. Furthermore amylases, are activated, that are involved in starch breakdown, while starch synthase (EC2.4.1.21) is down-regulated. This is surprising given that starch content is likely to be very low at this stage of fruit development (Souleyre et al., 2004). Some of these enzymes may still be required for breakdown of any residual starch to provide for the fruit energy requirements. Other enzymes such as the beta glucosidase 24-like enzyme may also have a function in the production of furanone VOCs (Orruño et al., 2001).

Changes in genes associated with phenylpropanoid metabolism confirmed previous work where strawberry fruit were stored at a much colder temperature of 0°C (Koyama et al., 2022) for a longer period of time (10 days). Genes encoding coniferyl-aldehyde dehydrogenase (EC:1.2.1.68), and beta-glucosidase (EC:3.2.1.21) were up-regulated in both studies while genes encoding cinnamyl alcohol dehydrogenase (EC:1.1.1.195) were down-regulated, and peroxidase (EC:1.11.1.7) genes were both up- and down-regulated during storage. However, genes for two further enzymes: shikimate O-hydroxycinnamoyl transferase (EC:2.3.1.133) and ferulate-5-hydroxylase (EC:1.14.-.-) were only up-regulated when fruit were exposed to 0°C for 10 days (Koyama et al., 2022) and not here where storage was at 8°C suggesting that genes for these enzymes are only activated at a colder storage temperature or a longer period of time. Conversely genes encoding other enzymes in this pathway, including cinnamoyl-CoA reductase (EC 1.2.1.44) were only down-regulated, and others including coniferyl-aldehyde dehydrogenase (EC 1.2.1.68), were only up-regulated here. Changes in the expression of these genes may be transient and hence only detectable earlier than 10 days of storage. They may also be related to the fruit wound response, since in this study fruit were halved, or may relate to the higher storage temperature which will accelerate postharvest degradation. The lack of change in abundance of most phenolic compounds analysed (Dhorajiwala et al., 2021) confirms this difference in temperature and time effect, although epicatechin levels did increase over the 5-day period. The down-regulation of farnesene and linalool pathways and peroxidases in the phenolpropanoid pathway that may be involved in terpene synthesis, again indicates a down-regulation of VOC production during storage although terpenes were a very minor component of the VOC profiles in this study.

Autophagy is associated with strawberry fruit ripening (Sánchez-Sevilla et al., 2021). Here expression of patterns of *ATG8* and *ATG4* genes were similar to those reported previously for the later stages of ripening (Sánchez-Sevilla et al., 2021) with *ATG8* expression increasing while *ATG4* expression fell between the white and red stages of ripening. Fruits here were harvested at the red ripe stage so the increase in *ATG8* expression could be related to continued ripening during storage which is known to occur in climacteric fruit such as peach (Sirangelo et al., 2022). However, the rise in expression between day 1 and day 5 of storage may also indicate that autophagy is increasing as the fruit tissue begins to run out of nutrients. During leaf and petal senescence *ATG8* expression is also up-regulated and is thought to be needed for nutrient remobilisation (Avila-Ospina et al., 2014). However, recently, a new autophagy-independent role for *ATG8* has been discovered in its interaction with *ABS3* to promote senescence (Jia et al., 2019). This has been hypothesised to be part of a nutrient sensing mechanism. In stored Chinese cabbage leaves *ATG8* expression also increased and was induced by methyl jasmonate and ABA (Zeng et al., 2022). Thus, in strawberry fruit an increase in *ATG8* expression may be indicative of an activation of autophagy when nutrients become severely limiting that may be either maintaining cell activity or pushing cells into senescence and cell death.

The overview of changes in TF gene expression indicate down-regulation of most of the TF families. This may relate to a general shut down of transcriptional activity during storage due to the cold treatment and also the progression of senescence. However, some TFs were up-regulated, and these may be important in mediating responses to the cold and the senescence process. Eighty-five TFs were recently identified as potential markers for storability (Min et al., 2020). Of these, only three: strawberry genes, with homology to *AIL5*, *RAP2.4* and *AGL29*, were differentially expressed here during storage. Min et al. (2020) identified the genes as changing in expression during ripening and between cultivars with different shelf-lives, so it is possible that many of these genes do not actually change in expression during storage.

Two TF families showing most up-regulation of gene expression were NAC and WRKY, both of which are closely associated to stress responses and senescence in other species (Nakashima et al., 2012; Jiang et al., 2017). From these the *WRKY32-like* gene showed up-regulation in the first day of storage. In Arabidopsis, *WRKY32* is part of a group of early cold responsive genes (Bakshi and Oelmüller, 2014) and in *F. vesca* this gene plays a positive regulatory role in pathogen resistance as well as a role in early fruit ripening (Wei et al., 2016; Zhou et al., 2016).

All seven of the TFs that were co-expressed with the AAT gene are most highly down-regulated during the first day of storage indicating that they may either be early cold responsive genes and/or responding to wounding. All three of the TFs co-expressed with AAT validated by real time PCR showed a similar pattern of change compared to the transcriptome data although for two of them changes were not significant, perhaps due to seasonal variation between the 2017 fruit used for the real time PCR and the 2014/2016 fruit used for the transcriptome. This may indicate transcription factors that are affected by pre-harvest fruit development. There was also variability across different fruit reflected in the error bars especially at day 0 which may reflect subtle differences in ripening or pre-harvest development. RF2a showed the most similar pattern of change compared to the *AAT* expression, however it was not identified as a potential regulator of *FaAAT* using PlantPAN3 software (**Supplementary Figure 5**). In contrast the ABF2-like gene is identified as a potential *FaAAT* regulator and is worthy of further investigation. In addition, the MYB21-like gene was previously identified in strawberry as *FaEOBII* as regulating the expression of *CINNAMYL ALCOHOL DEHYDROGENASE1* (*CAD1*) involved in terpenoid biosythensis (Medina-Puche et al., 2015). In this study no terpenoids were detected, however this may be variety-dependent. Verification *via* yeast-1-hybrid or ChIP seq and down-regulation in transgenic plants would be required to verify whether any or all of these seven TFs do indeed bind to the AAT promoter and if so whether the interaction is functional during fruit storage.

In conclusion this study provides an in-depth analysis of the effects of storage on halved strawberry fruit, of interest in the production of ready to eat fruit salads. It highlights the importance of studying VOC profiles across several seasons to assess the effects of storage on aroma, although esters remain the dominant VOC family. There is a core set of VOCs that is retained across seasons and these might provide useful quality markers during storage. The transcriptomic analysis shows that over a short storage period of 5 days at relatively low temperature thousands of genes change in expression probably relating to a combination of ripening, stress responses and senescence. Furthermore, co-expression analysis identifies seven TFs that may be regulators of *AAT* expression during storage and hence of interest in maintaining the ester profile.

## Data availability statement

The datasets presented in this study can be found in online repositories. The names of the repository/repositories and accession number(s) can be found in the article/**Supplementary Material**.

## Author contributions

AB, RD, CR, SD, ST, SJ, RL, LC, RA, NK, NS and HR conducted the experimental work and data analysis. AB, HR, CM and NS drafted the manuscript. CM, NS and HJR, designed the project. CM and HR acquired the funding. All authors contributed to the article and approved the submitted version.
