# Supplementary material for: Storage of halved strawberry fruits affects aroma, phytochemical content and gene expression, and is affected by pre-harvest factors
Source: Front Plant Sci. 2023 May 31;14:1165056. doi: 10.3389/fpls.2023.1165056 (PMC10264638; doi:10.3389/fpls.2023.1165056)
Supplement: Supplementary file 1 [file DataSheet_1.pdf]

Baldwin et al. Supplementary Tables and Figures

A

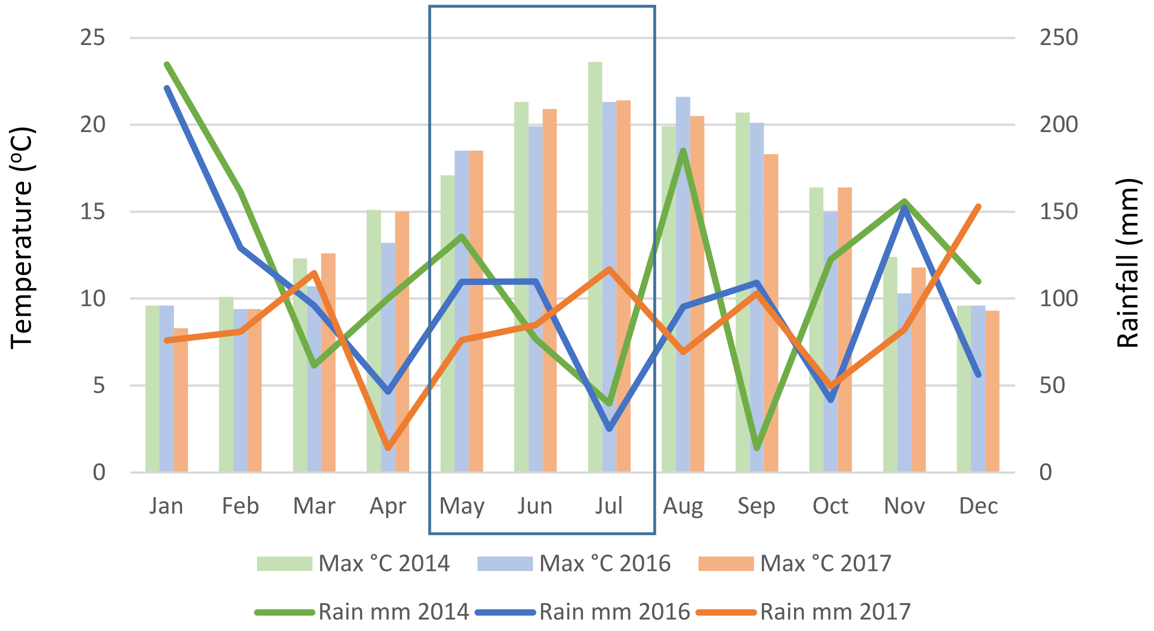

B

	temperature maximum difference across years (°C)	temperature minimum difference across years (°C)	difference in rainfall across years (mm)
Jan	1.3	1.2	158.6
Feb	0.7	2	80.4
Mar	1.9	3.2	53
Apr	1.9	2.2	85.8
May	1.4	0.9	59.4
Jun	1.4	0.6	33
Jul	2.3	0.6	91.6
Aug	1.7	1.8	115.7
Sep	2.4	2.8	94.8
Oct	1.4	2.3	80.8
Nov	2.1	2.8	73.6
Dec	0.3	0.9	96.7

Supplementary Figure 1. weather data for the three years in which strawberry material was collected: 2014, 2016 and 2017 from https://www.metoffice.gov.uk/pub/data/weather/uk/climate/stationdata/cardiffdata.txt for Cardiff Bute Park (-3.18728, 51.48783)
(A) comparison across the three years; (B) differences in minimum and maximum values for temperature and rainfall for each month across the three years (shaded to show greatest differences)

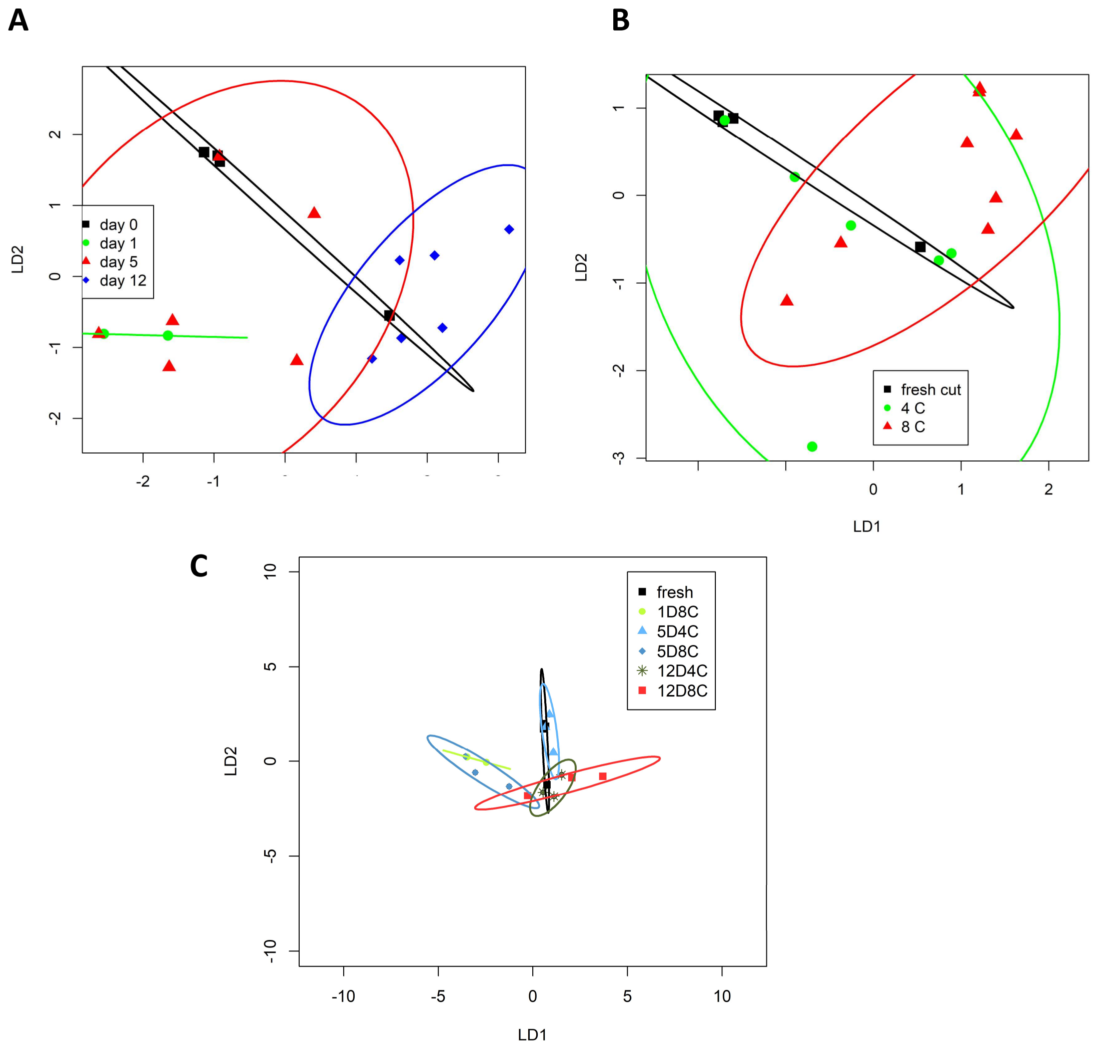

Supplementary Figure 2. Canonical Analysis of Principal coordinates related to time and temperature of storage based on all VOCs from 2016 season strawberry fruit stored for up to 12 days at 4 °C or 8°C analysed using TD-GC-TOF-MS: A CAP model was produced for strawberry samples **(A)** time of storage **(B)** temperature of storage **(C)** combined time (0, 1, 5 and 12 d) temperature. Each ellipse represents the 95% (SD) confidence interval. The plots use LD1 and LD2 with a percentage of correct classification of (A) 55.56 % and P =0.017 (n=3-6)); (B) 61.1 % and P = 0.027 (n=3-6) and (C) 38.89 % and P = 0.016 (n=2-3).

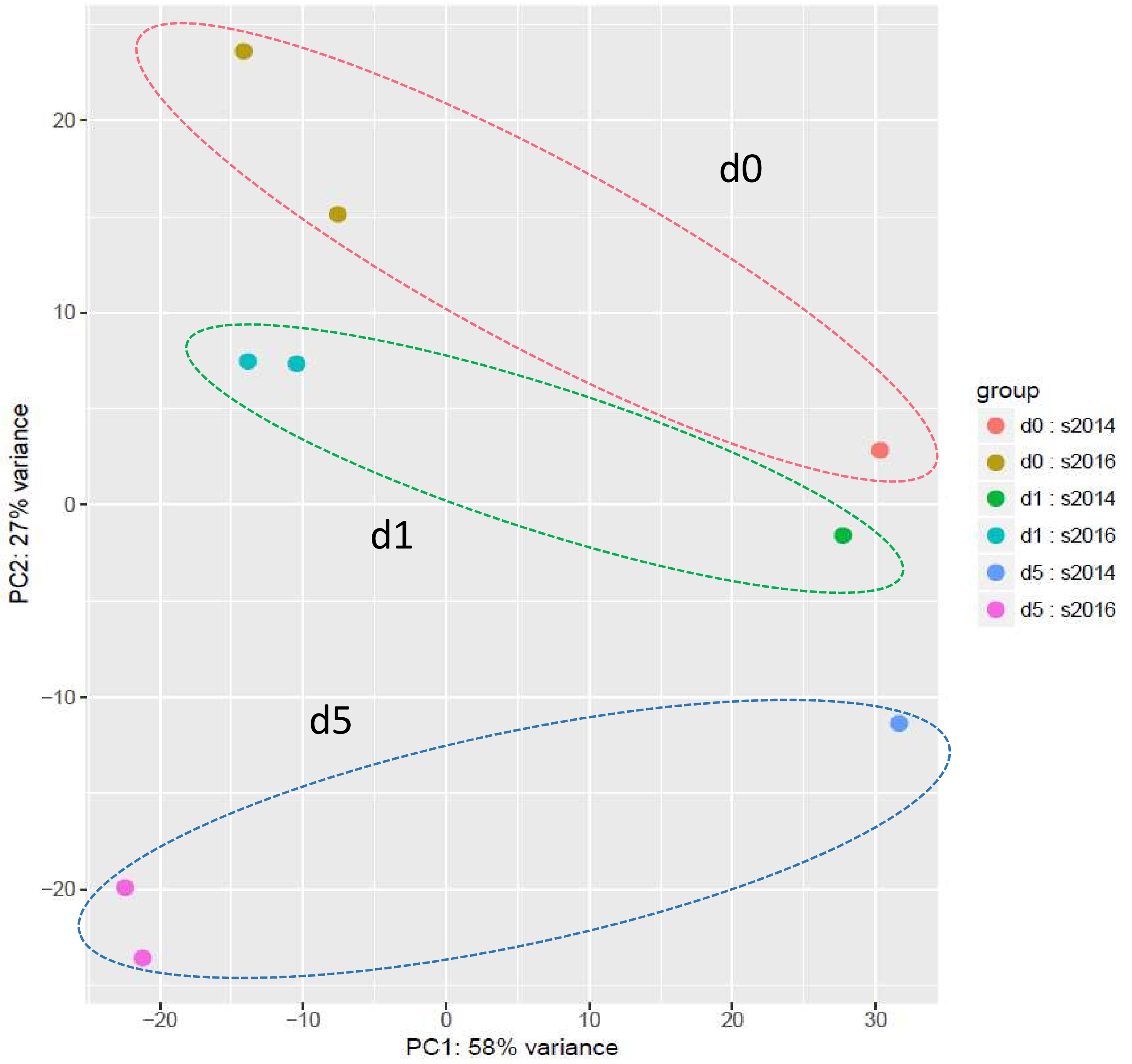

Supplementary Figure 3: PCA plot showing distribution of transcriptome feature counts of replicates from the mapped transcriptome three timepoints of strawberry storage (day 0 = d0, day 1 = da, day 5 = d5) and two seasons of harvest 2014 and 2016. Arbbitrary ellipses indicate the three replicates from each timepoint.

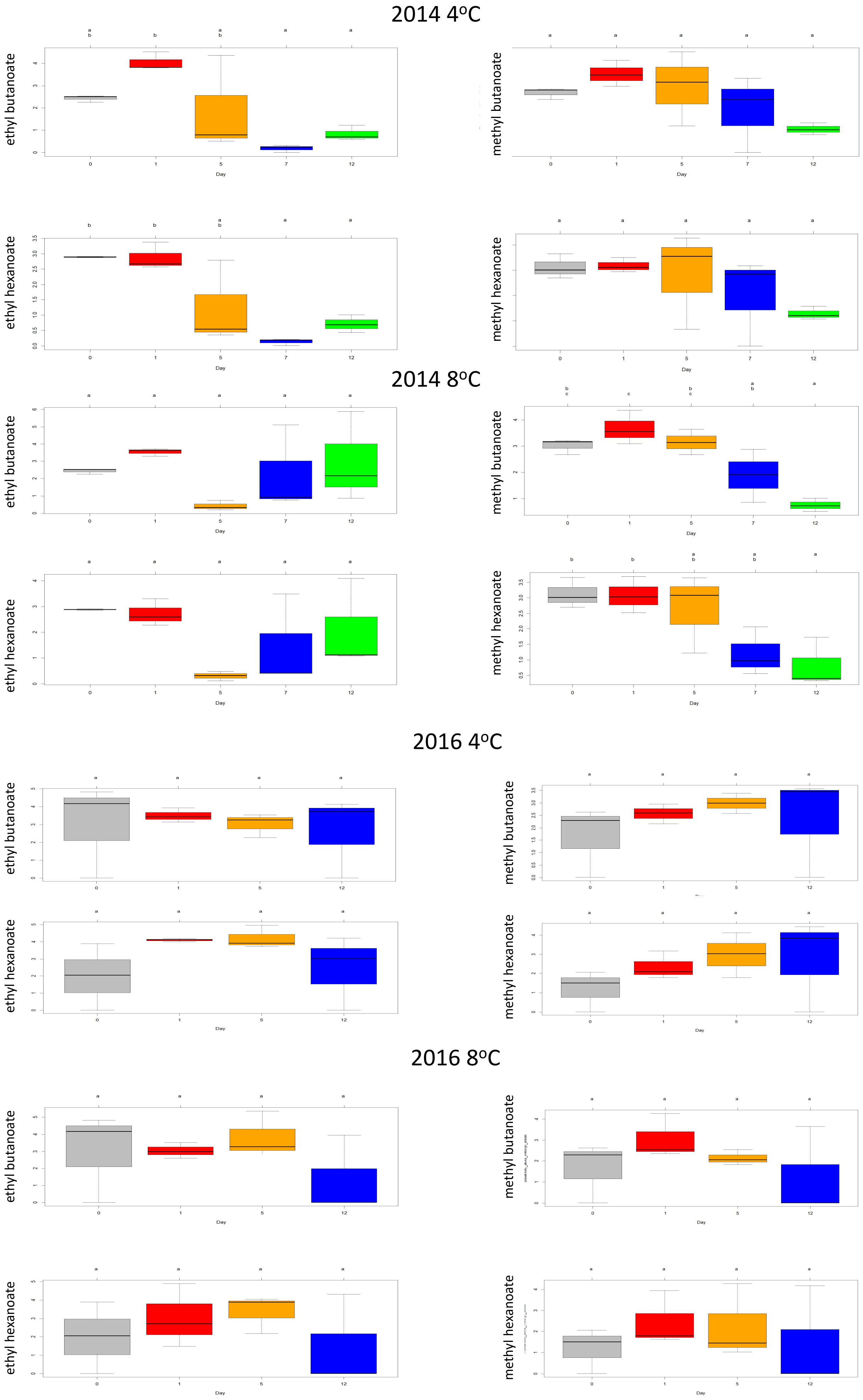

Supplementary Figure 4: relative abundance of ethyl butanoate, ethyl hexanoate, methyl butanoate and methyl hexanoate across days of storage, temperature of storage and fruit season. Different letters indicate significant differences (P <0.05, Tukey's test) within each sample set.

FaSAAT region 1000 bp upstream of ATG start codon:

ATTTTTTTTTCTTCGTCAAGTTCGGCAGCAGCATGGTACTAACTCTAATTTTTTCTATTTTTCTTTTCAGAATTTTG
ATGGCAAATTGAAGAACGCAAACACTTAGCCGCTTGGTTTCTTCCACATCTTGTACGTGGGATAAACCGCTTAA
ACCTAACCGTTTGATTTATTCCACGTCTTAGACGTGGGATAAATTGCTTAAAATGTTTAGTCTTTGTGTAGAACC
ACACAAACATTATCCTTGAAGATATCGAGGACATTGAAAGTGATAACGCCACCTCTCATCTAAGCTGGCTCATC
ATGATTTCACCTCTGTTAGCTTCATTGATGTTTCTTCAACGTGGTGGTTGATTCAATTATTTAATTTACCATAATTA
AATCAATAAGTCTTAATTGATCGATTTAATTATGACCGATAAGATATTTCACCGATTTGATTAAATTCTGACTTTG
ATTAAATTCACCGATTTGATTAAATTCAACGTGGTGGTTGATTCAATTATTTAATTTACCATAATTAAATCAATAA
GTCTTAATTGATCGATTTAATTATGACCGATAAGATATTTCACCGATTTGATTAAATTCTGACTTTGTTTTTGATA
ATCGATTTGATTTGATTGATGTCTGATTATCGATTAACATTTAATATTTGATTTTAGTCGGGATTGATGATTTTGA
TTTTATTAACATTTAACATTTAATATTAAATGTCTGATTATCAATTAATATTTGATTTTAGTTGGAATTGATGATTT
TGATTTGATTAACATTTAACATTTAATATCGACCGACTATTCACACGCCATCACGTGTCATTTCCGAATATTTTTC
AACTTTGCTGACTCACGAGCCACGTATGCAATTTATCGGGCTCATGTGTTGTATGAATAATTTAATGATTATTAT
TCTCATAAAATTTTATGCTTCAGTGTTCTATATATACCGCGTACCACATGTTTCATCAAGTGTCAGATAATACAGA
ACCTACTTCGCCAAA ATG

Fv region 1000 bp upstream of ATG start codon:
GTACTACTTTTGTTCTAGTTGATTTTCTTAATCCTTTGGCTTGTCCATATGCTTTCTAGAAGGAAACAAAGATTGT
CAAGGGTGACAACCCTCGTTTTTCATGGTGGGTACGTGAGCTTACTCTTTAATCAACTTTGATTGTCATCTCATT
ATGCAAAGCGGCAAATTTTTTTTCTTCGGCAAGTTCGGCAGCAGCATGGTACTAACTCTTTTTTCTATTTTTCTTT
TCAGAATTTTGGTGACAAATTGAAGAATGCGAACAACCTTTTGGTTTCTTTCACGTCTTGGATATGGGATAAAC
CGTTCAAAGTGTTTAGGTTTTATGTACCTTGACAGCAATTGAAGAACATAAACACCTAACACGCTTGATTTATTC
CACGTCTTAGACGTGGGATAAATCGCTTAAAGTGTTTAGGCTTTGCGTAGAC[GTATACTATTCCGCATGATGTT
TTCCAAGAGTTTCAAGAAGGACACAAACATCATCCTTGAAGATATCGAGGACATTGAAAGTGATGACACCACCT
CTCATCTAAGCTGGCTCATCATGATTTCATCTCTGTGAGCTTCATTGACGTATCTTCAACATGGTGGTTGATTTAA
TTATTTAATGTACCATAATTAAATCAATCAGTTTTAATTGATCGATTTAATTATGACCGATAAGGTATTTCACCAA
TTTAATTAAATTCCGACTTCCTTTTCGATAATCGATTTGATTTGATTGATGTCTGATTATCGATTGACATTTGATAT
TTAATTTTAGTCGGGATTGATGATTTTGTTGTTGACCGACTATTCACACGCCGCCACATGTTATTTCTGAAGACTT
TTCAACTTTGCTGACTTACGAGCCACGTATGCAATTTGTCAGATCCATGCGTTGTATGAATAATTTAATGATTAT
TATTCTCATTAAATTTTATGCTTCAGTTCTATATATATATACCGCGTACCACATGTTTCATCAAGTGTCAGATAAT
ATAGAACCTACCTCGCCAAAATG

Supplementary Figure 5 Potential binding sites for the ABI5-like TF on the AAT promoter region (highlighted in yellow); ATG start codon is highlighted in red. Based on analysis in PlantPAN3.0.

Supplementary Table 1: Primers used for RT-PCR.

Primer	Sequence (5' – 3')	Tm (°C)	Fragment size (bp)
FaActinF	GGGCCAGAAAGATGCTTATGTCGG	71.90	152
FaActinR	GGGCAACACGAAGCTATTGTAGAAG	67.40	
EF1F	GCCCATGGTTGTTGAAAACTTT	65.70	145
EF1R	GGCGCATGTCCCTCACA	67.60	
AATF	GAATCTGCAAGTTGCAAGTTCATAA	59.90	124
AATR	TCTTCTTCTAGATTCACCCACGC	61.50	
Gene31836F	GCCCTAACATCAGAGGTCCA	59.40	218
Gene31836R	TTTTCCTGCAGCTGTTGAGG	57.30	
Gene05263F	GCGGACGTTAGCCAAGAAAA	57.30	190
Gene05263R	CTCAACATCTTCTGCTGGCC	59.40	
Gene19256F	AGCGAGGAGATCAGGTGTTT	57.30	192
Gene19256R	GAACTCCATCTCCAGCTCCG	61.40	
Gene24852F	AGCTGAGAATGAGAGGCAAC	57.30	189
Gene24852R	TGGCGCGAGTACTGATGAAT	57.30	
Gene01680F	AGCAGCAATCACCTCAGACT	57.30	150
Gene01680R	ATTGATCTGACTCGGGCTTC	57.30	
Gene09110F	TTCTCTTCAACACTGCAAAGC	55.90	181
Gene09110R	GCCGTGTCACTGAAGTAAGC	59.40	
Gene02419F	ACCAGGGTTTCGATAATACAAGT	57.10	164
Gene02419R	ACATTCCTGCTTCCTTGCTC	57.30	

PRIMER	SEQUENCE (5' – 3')	TM (°C)	FRAGMENT SIZE (BP)
FaAATF	GAATCTGCAAGTTGCAAGTTCATAA	65.00	83
FaAATR	TCTTCTTCTAGATTCACCCACGC	65.40	
Fv02063F	TGGACCTCGATCAATTTGTGC	58.92	167
Fv02063R	TGGAGACCCTGCCTACCTTA	59.29	
Fv326286F	TGCCGAAGAGAAGTTTCCCT	58.94	196
Fv326286FR	GGTCACCCTAGCTCCAATACA	58.88	
Fv10176F	ACCGGATGCTAGCAGTGATT	59.27	208
FV10176R	TCGGGATTGCATCAGTGTCT	59.10	
Fv05512F	AAGGGCAGTTGGACGTGATA	59.02	213
FV05512R	GATACAGTCGCACGGTGAAC	59.01	
FaACTINF	GGGCCAGAAAGATGCTTATGTCGG	73.10	152
FaACTINR	GGGCAACACGAAGCTATTGTAGAAG	77.00	
PUV2	TTCCATGCTAATGTATTCAGAG	58.30	459
PUV4	ATGGTGGTGACGGGTGAC	64.70	

Supplementary Table 3: number of reads from transcriptomic analysis of stored strawberry fruit (Fragaria x ananassa cv. Elsanta)

Days of storage	Replicate 1 (M reads)	Replicate 2 (M reads)	Replicate 3 (M reads)
0	51.8	22.8	20.7
1	41.8	24.6	24.9
5	53.6	22.5	23.8
